# Evaluating AI Chatbots in Prosthodontics Education: A Quantitative MCQ‐Based Assessment

**DOI:** 10.1155/ijod/5985234

**Published:** 2026-09-17

**Authors:** Shankargouda Patil, Samuel Bybee, Venkata Suresh Venkataiah, Shareef Amor, Allahe Majidi, Shilpa Bhandi, Frank W. Licari

**Affiliations:** ^1^ College of Dental Medicine, Roseman University of Health Sciences, South Jordan 84095, Utah, USA, roseman.edu; ^2^ Clinical Sciences Department, Center of Medical and Bio-Allied Health Science Research, College of Dentistry, Ajman University, Ajman, UAE, ajman.ac.ae

**Keywords:** artificial intelligence, ChatGPT-5, clinical dentistry, multiple-choice questions, prosthodontics

## Abstract

**Purpose:**

Prosthodontics is a specialized branch of dentistry that encompasses a wide range of dental procedures, requiring advanced knowledge and expertise for both healthcare professionals. In this study, we assessed the performance of four widely accessible chatbots, ChatGPT‐5, Claude 4, Microsoft Copilot, and DeepSeek‐V3, using 150 clinical scenario‐based prosthodontics multiple‐choice questions (MCQs). Evaluating their performance is crucial to ensure reliability.

**Materials and Methods:**

A total of 150 questions compiled from Bootcamp in English were presented to the four chatbots. We then employed multiple evaluation criteria, such as bilingual evaluation understudy (BLEU), word error rate (WER), cosine semantic similarity, and Flesch reading ease (FRE) score, to provide a more comprehensive assessment of lexical similarity, error rate, semantic consistency, and readability.

**Results:**

Among the four chatbots, Microsoft Copilot (accuracy: 74%; confidence interval [CI]: 72.2%–76.2%) and ChatGPT‐5 (accuracy: 73%; CI: 71.5%–75.8%) demonstrated the best overall performance, achieving high accuracy at 95% CI. Particularly, ChatGPT‐5 showed the fewest word errors (WER: 7.86 [95% CI: 6.61–9.10]) and FRE readability of FRE: 20.22 (95% CI: 16.26–24.19) compared to Microsoft Copilot. In terms of alignment with reference outputs, based on the BLEU score, Claude 4 (0.0106 [95% CI: 0.0058–0.0154]) maintained higher scores compared to other analyzed chatbots. Collectively, ChatGPT‐5 produced a high level of observed accuracy with relatively fewer errors, moderate alignment with the reference answers, and favorable readability.

**Conclusion:**

This study suggests that ChatGPT‐5 demonstrated a favorable overall performance in prosthodontic clinical scenarios, with high observed accuracy, good textual and semantic alignment, relatively fewer errors, and favorable readability.

## 1. Introduction

Prosthodontics is a specialty of dentistry that deals with the rehabilitation of individuals with defects of missing teeth or maxillofacial structures that occur due to congenital conditions, disease, or trauma [1]. With substantial improvements in dentistry worldwide, the focus of prosthodontics has gradually shifted from removable dentures to fixed prostheses and implant‐supported restorations [2]. In particular, advances in implant procedures, the development of esthetic restorative materials, jaw‐tracking recorders, and sophisticated articulators have significantly improved prosthodontic treatment outcomes [3]. In recent years, research from academia and industry has led to the development of innovative technologies that have transformed dental practice [4]. The development of customized prostheses has enhanced the restoration of vital functions associated with the face, jaws, and neck, thereby improving physical health and psychological well‐being [5]. Consequently, prosthodontics has become an important component of the dental curriculum and requires practitioners and students to remain updated with rapidly evolving technologies, materials, digital workflows, and treatment protocols [6]. Given the expanding prosthodontic innovations and the complexity of clinical decision‐making, there is a need for intelligent digital tools that can provide timely, accurate, and context‐specific information [7].

Recent technology has revolutionized both clinical practice and dental education [8]. Among these advances, artificial intelligence (AI) is one of the most significant domains, which is a rapidly growing area in healthcare applications, including diagnosis, treatment planning, patient management, and education [9]. AI chatbots are computerized systems that are trained on huge amounts of textual data and can understand, process, and generate human‐like responses [10]. These systems use state‐of‐the‐art machine learning and natural language processing (NLP) techniques to imitate human language patterns and interact in conversation [11]. In dentistry, AI chatbots have demonstrated significant potential in enhancing student learning, facilitating knowledge acquisition, supporting teleconsultation, and assisting clinical decision‐making [12]. In particular, chatbot‐based multiple‐choice question (MCQ) learning has emerged as a promising educational approach, enabling learners to gain knowledge, strengthen clinical reasoning skills, and obtain immediate feedback in a self‐directed learning environment [13]. Several studies have investigated the application of chatbots in prosthodontics. Chau et al. [14] assessed three AI chatbots’ responses to MCQs in prosthodontics and restorative dentistry, focusing primarily on response accuracy and quality. Ates and Bulut [15] compared the performance of advanced LLMs with prosthodontic residents using standardized multiple‐choice examinations, suggesting that these tools could be used as an additional teaching aid. Similarly, Bulut et al. [16] assessed the performance of multiple AI chatbots on prosthodontic examination questions and demonstrated significant variations in accuracy among different models. Furthermore, Akpinar [17] evaluated the accuracy of ChatGPT 4.0, OpenEvidence, and MediSearch in their responses to questions about the All‐on‐Four dental implant concept with a 5‐point Likert scale. Although previous studies have demonstrated the potential of AI chatbots in prosthodontics, most investigations have primarily focused on response accuracy and overall answer quality. Comprehensive assessments encompassing performance, lexical, semantic, readability, and statistical metrics remain limited. Therefore, a multidimensional assessment is required to understand the clinical utility, reliability, and limitations of AI chatbots in prosthodontics.

This study evaluated the performance of four widely used chatbots (ChatGPT‐5, Claude 4, Microsoft Copilot, and DeepSeek‐V3) in answering prosthodontics‐based clinical scenario questions. The generated responses were evaluated using performance metrics, NLP measures, and readability assessments, allowing for a comprehensive comparison of their accuracy, linguistic similarity, semantic coherence, and overall relevance. Therefore, our study shows promise for chatbots as useful additional digital tools for prosthodontic education, augmenting traditional teaching methods and improving learning support for dental students and practitioners.

## 2. Methods

### 2.1. Study Design and Dataset Preparation

This cross‐sectional comparison investigation was performed following the STROBE (Strengthening the Reporting of Observational Studies in Epidemiology) principles (File S1). Two authors (Samuel Bybee and Venkata Suresh Venkataiah) extracted 200 prosthodontic clinical scenario‐based MCQs and their ground truth answers from the Integrated National Board Dental Examination (INBDE) Bootcamp repository (https://bootcamp.com; accessed August 2025), a widely used resource for dental education and clinical skill assessment. These MCQs were screened by two authors (Shareef Amor and Allahe Majidi) according to predecided inclusion and exclusion criteria. Eligible MCQs were prosthodontically relevant, patient‐based prosthodontic situations demanding clinical reasoning, and featured four response choices with one correct answer. Questions were excluded if they did not involve a clinical scenario or higher‐order clinical reasoning, did not have four answer options with one correct answer, were not directly related to prosthodontics, were duplicates or had substantial overlap with other questions, or had visual content (e.g., radiographs, clinical photographs, diagrams, or prosthetic images) so that text‐only questions could be uniformly evaluated across chatbot platforms. A total of 183 MCQs were found eligible following the screening. Two authors (Samuel Bybee and Frank W. Licari) categorized the MCQs into the following predetermined domains in prosthodontics: complete dentures, removable partial dentures, fixed prosthodontics, implant prosthodontics, occlusion, impression techniques, esthetic dentistry, and oral rehabilitation/maxillofacial prosthodontics. Each MCQ was allocated to a single primary area according to the main clinical focus. The corresponding author (Shankargouda Patil) assessed the categorized MCQs; in cases of overlap between multiple domains, a consensus was obtained with other investigators to assign to the single most dominant domain, confirmed the distribution of domains, and finalized a dataset of 150 MCQs for analysis.

### 2.2. Selection of Chatbots and Testing Workflow

Four open‐source AI chatbots: ChatGPT‐5 (OpenAI, https://chatgpt.com), Claude 4 (https://claude.ai), Microsoft Copilot (https://CoPilot.microsoft.com), and DeepSeek‐V3 (https://www.DeepSeek-V3.com) were used for evaluation. These chatbots were selected based on their wide user accessibility, high popularity, clinical reasoning capabilities, integration of state‐of‐the‐art technologies, and overall relevance [18]. An identical question format was employed across all chatbots to maintain consistency and ensure a standardized evaluation process: “For the following MCQs, please pick one correct answer with an explanation.” The responses were processed through a two‐step CSV‐based workflow that included (1) question number, ground truth, and chatbot‐generated options and (2) compiling the full‐text ground‐truth answers alongside the chatbot‐generated explanations. MCQs were presented domain‐wise using a standardized prompt, instructing the chatbot to select one correct answer with an explanation. Each domain was treated as an independent session, with all context‐related features disabled to avoid any carryover effects between domains. All chatbot models were evaluated under their default configurations without altering any generation parameters. The entire analysis was performed using a local NVIDIA GPU setup in combination with Google Colab’s cloud‐based environment, employing Jupyter Notebook (version 6.5.7) and Python (version 3.11.13) (https://colab.research.google.com) [19].

### 2.3. Chatbot Performance Analysis

Python 3.11.13 with pandas (v2.1.4), regex (v2024.5.15), scikit‐learn.metrics (v1.6.1), matplotlib.pyplot (v3.10.0), seaborn (v0.13.2), numpy (v1.22.4), rouge (v0.1.2), and jiwer (v3.1.0) libraries were used for all data processing, including regular expression, graphical visualization, and statistical testing. The accuracy with 95% confidence intervals (CIs) for each chatbot was determined by calculating the percentage of questions answered correctly out of the total number of questions assessed, providing a measure of overall performance [20]. Next, the readability, semantic correctness, and response quality of the 150 MCQs produced by the four chatbots (ChatGPT‐5, Claude 4, Microsoft Copilot, and DeepSeek‐V3) were examined. The n‐gram overlap with reference outputs was evaluated using the bilingual evaluation understudy (BLEU) score [21]. While the word error rate (WER) quantifies textual inconsistencies, [22] cosine semantic similarity measures conceptual alignment [23] and the Flesch reading ease (FRE) score assesses the readability of the generated explanations [24].

### 2.4. Statistical Analysis

All statistical analyses were performed using Python 3.11.13. The quantitative data, including chatbot accuracy and text‐based performance metrics, were summarized as average values with corresponding 95% CIs for comparison between the evaluated chatbot models. A one‐way analysis of variance (ANOVA) [25] was performed to assess the overall statistical differences between the chatbots for the BLEU score, WER, cosine semantic similarity, and FRE score. Further, the Mann–Whitney *U* test [26] pairwise comparisons between chatbot models were conducted to identify the contributing group for the observed differences. Statistical significance was considered at a two‐sided *p*‐value ≤ 0.05.

## 3. Results

### 3.1. Performance Analysis of Four Chatbots

**Figure 1:**
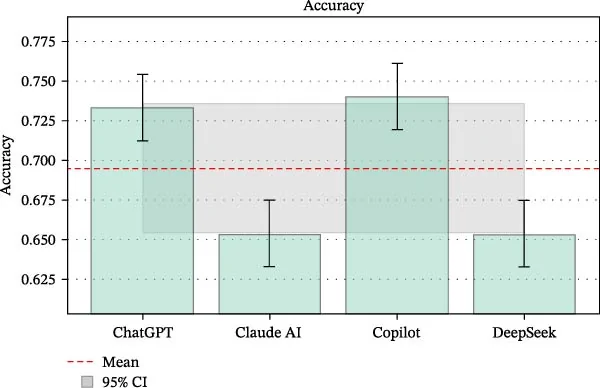
Performance metrics such as accuracy were calculated for ChatGPT‐5, Claude 4, Microsoft Copilot, and DeepSeek‐V3 with 95% confidence intervals.

The screened 150 MCQs were categorized into major domains of prosthodontics to ensure a balanced representation of topics and a reliable distribution of questions across the discipline. The final dataset comprised 19 MCQs each from complete dentures, removable partial dentures, fixed prosthodontics, implant prosthodontics, occlusion, and impression techniques, while esthetic dentistry and oral rehabilitation/maxillofacial prosthodontics contributed 18 MCQs, respectively. Subsequently, the performances of four chatbots (ChatGPT‐5, Claude 4, Microsoft Copilot, and DeepSeek‐V3) were evaluated through these 150 MCQs with their pre‐established ground truth sourced from the INBDE Bootcamp materials. For each chatbot, performance was assessed based on accuracy with the corresponding 95% CIs. Among the four chatbots, Microsoft Copilot showed a higher accuracy of 74% (95% CI: 72.2%–76.2%), followed closely by ChatGPT‐5 with an accuracy of 73% (95% CI: 71.5%–75.8%). Claude 4 and DeepSeek‐V3 demonstrated comparatively lower but similar observed accuracies of 65% (95% CI: 63.7%–67.0% and 63.5%–67.0%, respectively). Overall, Microsoft Copilot and ChatGPT‐5 showed similar and numerically higher accuracy in correctly answering the prosthodontic clinical scenario‐based MCQs (Figure 1; Table 1).

**Table 1:** Accuracy (%) and 95% confidence intervals of the evaluated AI chatbots in answering prosthodontic clinical scenario‐based MCQs.

SI. No.	Chatbot	Accuracy (%)	95% confidence interval
1	ChatGPT‐5	73	71.5–75.8
2	Claude 4	65	63.7–67.0
3	Microsoft Copilot	74	72.2–76.2
4	DeepSeek‐V3	65	63.5–67.0

### 3.2. BLEU Score Lexical Similarity Analysis of Chatbots

**Figure 2:**
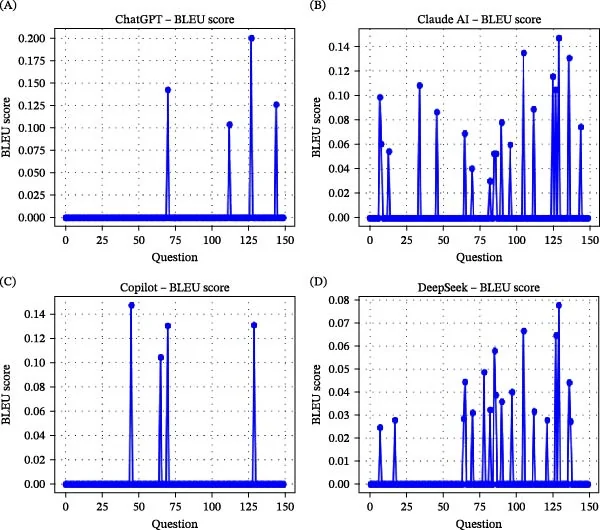
The BLEU scores for (A) ChatGPT‐5, (B) Claude 4, (C) Microsoft Copilot, and (D) DeepSeek‐V3 indicate the degree of alignment with the reference text.

Based on lexical similarity, Claude 4 achieved the highest BLEU score (0.0106 [95% CI: 0.0098–0.0114]), followed by DeepSeek‐V3 (0.0050 [95% CI: 0.0046–0.0054]), ChatGPT‐5 (0.0038 [95% CI: 0.0032–0.0044]), and Microsoft Copilot (0.0034 [95% CI: 0.0029–0.0039]) (Table 2). The ANOVA revealed a statistically significant difference in BLEU scores among the evaluated chatbot models with a *p*‐value ≤ 0.0253 (Table 3). Notably, the absolute BLEU scores of all the tested chatbots were low, suggesting a significant overlap in the lexical similarity with the reference responses (Figure 2). Pairwise comparisons using the Mann–Whitney *U* test demonstrated significant differences between the evaluated chatbot models across several performance metrics (Table 4). For the BLEU score, ChatGPT‐5 differed significantly from Claude 4 (*p* = 0.0072) and DeepSeek‐V3 (*p* = 0.0039), but not from Microsoft Copilot (*p* = 0.3819). Likewise, significant differences were observed between Claude 4 and Microsoft Copilot (*p* = 0.0011), and between Microsoft Copilot and DeepSeek‐V3 (*p* = 0.0005), whereas the difference between Claude 4 and DeepSeek‐V3 was not statistically significant (*p* = 0.2077).

**Table 2:** Mean BLEU scores and WER values of the evaluated AI chatbots.

SI. No.	Chatbots	BLEU score mean (95% CI)	WER mean (95% CI)
1	ChatGPT‐5	0.0038 (95% CI: 0.0000–0.0076)	7.8551 (95% CI: 6.61–9.10)
2	Claude 4	0.0106 (95% CI: 0.0058–0.0154)	21.7515 (95% CI: 18.86–24.64)
3	Microsoft Copilot	0.0034 (95% CI: 0.0001–0.0067)	9.6828 (95% CI: 8.32–11.04)
4	DeepSeek‐V3	0.0050 (95% CI: 0.0027–0.0073)	34.3975 (95% CI: 30.52–38.27)

*Note:* Error bars indicate 95% confidence intervals.

**Table 3:** ANOVA test results for four LLMs across four evaluation metrics.

SI. No.	Scoring metrics	Calculated *p*‐Value
1	BLEU score	0.0253 ^∗^
2	Word error rate	<0.0001 ^∗^
3	Cosine similarity	0.0012 ^∗^
4	Flesch readability ease	<0.0001 ^∗^

^∗^ *p*‐Value < 0.05, statistically significant.

**Table 4:** Pairwise comparison of NLP and readability metrics among chatbots.

Comparison	BLEU score (*p*‐Value)	WER (*p*‐Value)	Cosine similarity (*p*‐Value)	Flesch reading ease (*p*‐Value)
ChatGPT‐5 vs. Claude 4	0.0072 ^∗^	<0.0001 ^∗^	0.0895	0.3223
ChatGPT‐5 vs. Microsoft Copilot	0.3819	0.0117 ^∗^	0.0642	0.0136 ^∗^
ChatGPT‐5 vs. DeepSeek‐V3	0.0039 ^∗^	<0.0001 ^∗^	0.8452	<0.0001 ^∗^
Claude 4 vs. Microsoft Copilot	0.0011 ^∗^	<0.0001 ^∗^	0.0004 ^∗^	0.0559
Claude 4 vs. DeepSeek‐V3	0.2077	<0.0001 ^∗^	0.0679	<0.0001 ^∗^
Microsoft Copilot vs. DeepSeek‐V3	0.0005 ^∗^	<0.0001 ^∗^	0.0394 ^∗^	<0.0001 ^∗^

^∗^ *p*‐Value < 0.05, statistically significant.

### 3.3. WER of Chatbots

**Figure 3:**
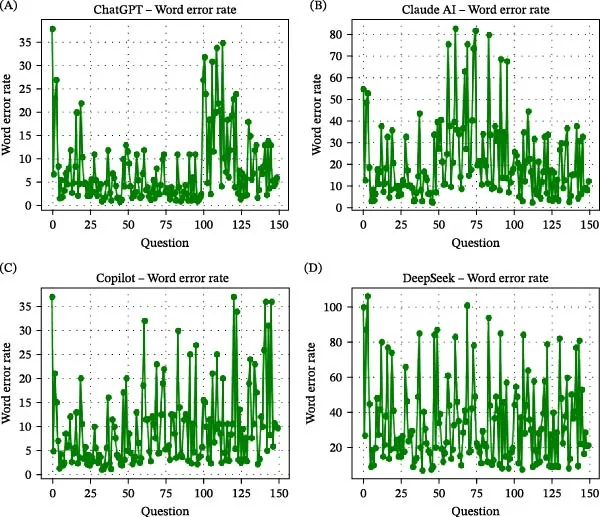
The WER scores for (A) ChatGPT‐5, (B) Claude 4, (C) Microsoft Copilot, and (D) DeepSeek‐V3, pointing out their word errors.

Based on the WER analysis, lower WER was noticed for ChatGPT‐5 (7.86 [95% CI: 6.61–9.10]) and Microsoft Copilot (9.68 [95% CI: 8.32–11.04]), indicating relatively fewer word‐level differences with the reference text (Table 2). While DeepSeek‐V3 (34.40 [95% CI: 30.52–38.27]) and Claude 4 (21.75 [95% CI: 18.86–24.64]) had higher WER values (Figure 3), these reflect the lexical differences from the reference text. Further, the statistical analysis based on ANOVA demonstrated a significant difference in WER among the chatbot models (*p* < 0.001) (Table 3). Additionally, the pairwise comparisons based on the Mann–Whitney *U* test indicate distinct word‐level performance among the evaluated chatbot models with statistical significance across the models (Table 4).

### 3.4. Cosine Semantic Similarity Consistency

**Figure 4:**
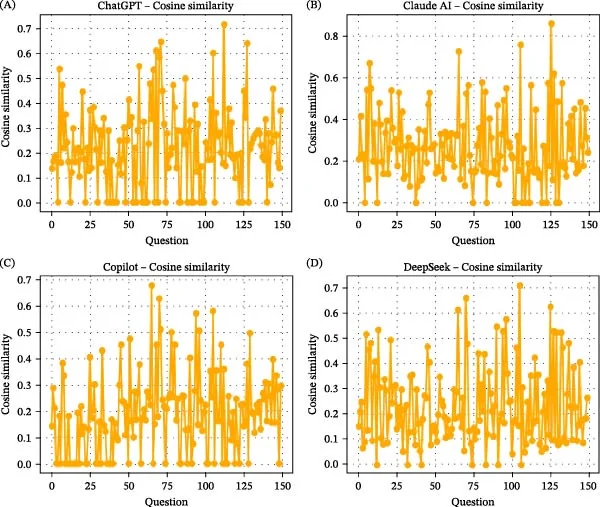
The cosine similarity scores for (A) ChatGPT‐5, (B) Claude 4, (C) Microsoft Copilot, and (D) DeepSeek‐V3 indicating their semantic similarity.

In cosine semantic similarity analysis, Claude 4 outperformed the other chatbots with the score of 0.2637 (95% CI: 0.2360–0.2914), followed by DeepSeek‐V3 with 0.2327 (95% CI: 0.2076–0.2578), ChatGPT‐5 with 0.2214 (95% CI: 0.1952–0.2476), and Microsoft Copilot with 0.1892 (95% CI: 0.1645–0.2139), suggesting better semantic similarity to the reference responses (Figure 4 and Table 5). Further, the differences in cosine semantic similarity among the chatbot models were statistically significant according to ANOVA with a *p*‐value ≤ 0.0012 (Table 3). Further, the comparative pairwise analysis suggests significant differences between Claude 4 and Microsoft Copilot (*p* = 0.0004), and between Microsoft Copilot and DeepSeek‐V3 (*p* = 0.0394). However, no significant differences were found between ChatGPT‐5 and Claude 4 (*p* = 0.0895), ChatGPT‐5 and Microsoft Copilot (*p* = 0.0642), ChatGPT‐5 and DeepSeek‐V3 (*p* = 0.8452), or Claude 4 and DeepSeek‐V3 (*p* = 0.0679) (Table 4).

**Table 5:** Mean cosine similarity and Flesch reading ease (FRE) scores of the evaluated AI chatbots.

SI. No.	Chatbots	Cosine similarity mean (95% CI)	FRE score mean (95% CI)
1	ChatGPT‐5	0.2214 (95% CI: 0.1952–0.2476)	20.2230 (95% CI: 16.26–24.19)
2	Claude 4	0.2637 (95% CI: 0.2360–0.2914)	18.3591 (95% CI: 15.73–20.99)
3	Microsoft Copilot	0.1892 (95% CI: 0.1645–0.2139)	13.9823 (95% CI: 11.12–16.84)
4	DeepSeek‐V3	0.2327 (95% CI: 0.2076–0.2578)	35.3620 (95% CI: 33.21–37.52)

*Note:* Error bars indicate 95% confidence intervals.

### 3.5. FRE Score for Readability

**Figure 5:**
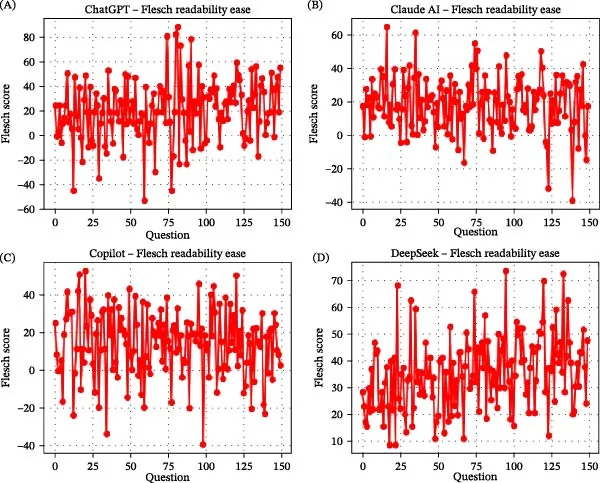
The readability scores for (A) ChatGPT‐5, (B) Claude 4, (C) Microsoft Copilot, and (D) DeepSeek‐V3, based on FRE scores.

The readability assessment with FRE shows DeepSeek‐V3 and ChatGPT‐5 with higher FRE scores (35.36 [95% CI: 33.21–37.52]) and 20.22 (95% CI: 16.26–24.19), respectively, than Claude 4 (18.36 [95% CI: 15.73–20.99]) and Microsoft Copilot (13.98 [95% CI: 11.12–16.84]) (Figure 5; Table 5) with a statistical significance of *p* < 0.001 (Table 3). Notably, the obtained FRE scores were in the range of the difficult and very difficult readability categories, indicating that the responses generated required a relatively high reading level and were not easy to comprehend. Further, the Mann–Whitney *U* test shows significant differences between ChatGPT‐5 and Microsoft Copilot (*p* = 0.0136), ChatGPT‐5 and DeepSeek‐V3 (*p*  < 0.001), Claude 4 and DeepSeek‐V3 (*p*  < 0.001), and Microsoft Copilot and DeepSeek‐V3 (*p*  < 0.001). In contrast, no statistically significant differences were detected between ChatGPT‐5 and Claude 4 (*p* = 0.3223) or between Claude 4 and Microsoft Copilot (*p* = 0.0559) (Table 4).

## 4. Discussion

AI chatbots have the potential to transform several areas of dentistry. In particular, AI algorithms can increase the precision of radiographic interpretation and disease screening, assisting in the early identification of oral diseases overlooked by physicians [27]. The rapid growth of AI chatbots has great potential for patient care and medical education, especially in terms of improving patient interactions and offering easily accessible educational resources [28]. For these applications, the precision and quality of the generated responses are crucial. Recent studies have explored the performance of AI chatbots in implant dentistry and implant‐supported treatment concepts. For instance, Revilla‐León et al. [29] pointed out that ChatGPT provided highly accurate answers for 50 MCQs in the field of implant dentistry. Also, Binaljadm et al. [30] evaluated the responses of three large language model‐based chatbots such as GPT‐3.5, Gemini, and Microsoft Copilot to 20 frequently asked dental questions.

Herein, this study extends by comparing the performance of ChatGPT‐5, Claude 4, Microsoft Copilot, and DeepSeek‐V3 on prosthodontics MCQs by assessing the accuracy of responses, similarity with reference text, error profiling, and readability analyses for better prosthodontic education. ChatGPT‐5, Claude 4, Microsoft Copilot, and DeepSeek‐V3 were chosen because of their wide adoption, public availability, representation of current LLM technologies, and demonstrated effectiveness in various dental applications. Eraslan et al.[31] found that Microsoft Copilot was the most accurate in answering prosthodontic questions. Similarly, Alqahtani [32] reported statistically significant differences in the performance of Claude 4, ChatGPT‐4o, and DeepSeek‐V3 in dental occlusion, implying that the performance of chatbots varies according to the complexity and domain specificity of the task. Additionally, Altos et al. [33] found that GPT‐5 outperformed Claude and DeepSeek‐V3 in case‐based dental MCQs with medically compromised patients. Furthermore, Umer et al. [34] emphasized the growing role of LLMs in dental education, scientific writing, patient communication, and clinical decision support.

A list of 200 MCQs was collected from the INBDE Bootcamp materials, which cover a broad range of clinical scenarios relevant to prosthodontics. INBDE Bootcamp is a comprehensive online study resource for the INBDE. It has 3000+ board‐style MCQs, case‐based clinical scenarios, simulation exams, video lectures, and detailed explanations according to the INBDE blueprint. The collected questions were screened according to predefined inclusion criteria, resulting in a final dataset of 150 prosthodontics‐based clinical scenario MCQs for chatbot evaluation. These 150 MCQs were evaluated based on metrics like accuracy, BLEU score, WER, cosine similarity, and FRE scores to measure their performance for each chatbot. Microsoft Copilot demonstrated the highest observed accuracy among the models evaluated, with 74% of the responses answered correctly, followed closely by ChatGPT‐5, which achieved an accuracy of 73%. Claude 4 and DeepSeek‐V3 each demonstrated an accuracy of 65%. The relatively high observed accuracy of Microsoft Copilot may be attributed to its integration of large language models with retrieval‐augmented generation and web‐based information retrieval, which may facilitate access to current and contextually relevant information. Similarly, the comparable accuracy observed for ChatGPT‐5 may reflect its advanced reasoning and contextual understanding capabilities, which may enable effective interpretation and response to complex biomedical and prosthodontic concepts. These results are consistent with those of Salman et al. [35], who reported Microsoft Copilot and ChatGPT as among the highest‐performing AI chatbots for generating accurate responses to dental questions. The performance differences between the evaluated chatbot models suggest the role of their underlying large language model architectures, training methods, reasoning capabilities, and optimization strategies. Although Microsoft Copilot and ChatGPT‐5 had better accuracy, response correctness alone does not fully reflect the quality or educational value of the chatbot‐generated content. Therefore, additional evaluations based on BLEU, WER, cosine semantic similarity, and FRE score were performed to provide a more comprehensive assessment of the linguistic quality and applicability of the generated responses.

The BLEU score measures the overlap of n‐grams between the chatbot‐generated responses and reference answers to determine their lexical similarity. Analysis showed that Claude 4 and DeepSeek‐V3 had higher BLEU scores, indicating closer lexical alignment with the reference text, whereas the lower BLEU scores of ChatGPT‐5 and Microsoft Copilot suggested that these models produced correct responses in different words instead of closely matching the reference answers (Table 2). Therefore, lower BLEU scores should be interpreted as reduced lexical similarity rather than poorer response quality [36]. Interestingly, the pairwise comparison did not show a statistically significant difference in BLEU scores between ChatGPT‐5 and Microsoft Copilot (*p* = 0.3819), indicating that both models exhibited a similar pattern of lexical similarity despite their high accuracy. This result indicates that both chatbots use similar strategies for language generation with clinically accurate responses. Likewise, ChatGPT‐5 achieved a significantly lower WER (*p* = 0.0117) (7.86 [95% CI: 6.61–9.10]) than Microsoft Copilot (9.68 [95% CI: 8.32–11.04]), suggesting closer lexical agreement with the reference text and superior word‐level accuracy. Moreover, the cosine semantic similarity score of ChatGPT‐5 (0.2214 [95% CI: 0.1952–0.2476]) and Microsoft Copilot (0.1892 [95% CI: 0.1645–0.2139]) were not significantly different (*p* = 0.0642) which suggest that the conceptual meaning of the reference responses generated by the ChatGPT‐5 and Microsoft Copilot were similar in semantic consistency with the reference text, though the cosine similarity scores are slightly different. Finally, readability assessment through the FRE score revealed a significant difference between ChatGPT‐5 and Microsoft Copilot (*p* = 0.0136). ChatGPT‐5 achieved a higher FRE score (20.22 [95% CI: 16.26–24.19]) than Microsoft Copilot (13.98 [95% CI: 11.12–16.84]), indicating that its responses were comparatively easier to read than those of Microsoft Copilot. Nevertheless, all four chatbots generated responses within the “difficult” to “very difficult” readability categories, suggesting that the content is more appropriate for dental students and professionals than for patients or the general public. Overall, these findings indicate that ChatGPT‐5 demonstrated a favorable balance of accuracy, reliability, and readability among the evaluated models. Our results are consistent with those of Durmazpinar and Ekmekci [37] and Nguyen et al. [38], who also noted ChatGPT’s low error rates, high accuracy, and strong alignment with reference texts. Therefore, this study suggests that ChatGPT‐5 demonstrated a favorable overall performance across various clinical scenarios in prosthodontic dentistry, indicating its potential utility in dental education, knowledge acquisition, and clinical decision‐making. However, the study holds a few limitations, such as (1) using specific large language model versions, (2) a predefined set of MCQs and evaluation metrics, and (3) variability of responses across repeated generations. Future studies should include repeated response analyses, broader clinical question formats, and updated chatbot versions to provide a more comprehensive evaluation of AI‐assisted learning tools in prosthodontics.

## 5. Conclusion

In conclusion, this study evaluated the effectiveness of four popular AI chatbots, ChatGPT‐5, Claude 4, Microsoft Copilot, and DeepSeek‐V3, using 150 prosthodontic clinical scenario‐based MCQs. Based on the overall performance across the evaluated metrics, ChatGPT‐5 demonstrated a relatively balanced performance across accuracy, lexical similarity, semantic consistency, and readability. Our results suggest that AI chatbots may serve as valuable adjuncts to prosthodontic education by facilitating knowledge acquisition, self‐directed learning, and exam preparation. The findings, however, should be interpreted with caution and should not be directly applied to real‐world clinical decision‐making as the assessment was limited to MCQ tests. Further research with complex clinical scenarios, expert review, and empirical testing is needed to assess the educational and clinical performance of these AI chatbots in prosthodontics.

## Author Contributions

Conceptualization, methodology, analysis, investigation, data curation, writing – original draft preparation: Shankargouda Patil, Samuel Bybee, Venkata Suresh Venkataiah, and Shareef Amor. Software, validation, visualization, resources, writing – review and editing: Allahe Majidi, Shilpa Bhandi, and Frank W. Licari.

## Funding

The authors have nothing to report.

## Disclosure

All authors have read and agreed to the published version of the manuscript.

## Ethics Statement

The authors have nothing to report.

## Conflicts of Interest

The authors declare no conflicts of interest.

## Supporting Information

Additional supporting information can be found online in the Supporting Information section.

## Supporting information


**Supporting Information** File S1: STROBE (Strengthening the Reporting of Observational Studies in Epidemiology) guidelines.

## Data Availability

The datasets used and analyzed during the current study are available from the corresponding author upon reasonable request.
