# Supplementary material for: Evaluating AI Chatbots in Prosthodontics Education: A Quantitative MCQ‐Based Assessment
Source: Int J Dent. 2026 Sep 17;2026:5985234. doi: 10.1155/ijod/5985234 (PMC13586676; doi:10.1155/ijod/5985234)
Supplement: Supplementary file 1 [file IJOD-2026-5985234-s001.docx]

**Supplementary file 1: STROBE (Strengthening the Reporting of Observational Studies in Epidemiology) guidelines**

|  | Item No | Recommendation |
| --- | --- | --- |
| **Title and abstract** | 1 | (*a*) Title and Abstract – Comparative evaluation study |
|  |  | (*b*) Abstract – Structured summary of objectives, methods, results, and conclusions. |
| Introduction | | |
| Background/rationale | 2 | AI chatbots are increasingly being used in healthcare and dental education, and this has resulted in a need to evaluate the accuracy, reliability, semantic consistency, and educational utility of these tools in prosthodontics. |
| Objectives | 3 | To compare the performance of the models (ChatGPT, Claude, Copilot, and DeepSeek) on 150 prosthodontic clinical scenario-based MCQs using accuracy, lexical similarity, semantic similarity, readability, and statistical evaluation metrics. |
| Methods | | |
| Study design | 4 | Comparative evaluation study of four AI chatbots using 150 prosthodontic clinical scenario-based MCQs. |
| Setting | 5 | MCQ Preparation. MCQs were compiled from the Bootcamp repository in August 2025 and evaluated using selected chatbots under standardized testing conditions. |
| Participants | 6 | N/A – No human participants. A curated dataset of 150 prosthodontic MCQs was randomly selected and expert-validated. |
| Variables | 7 | Outcome Measures. Accuracy, BLEU score, Word Error Rate (WER), cosine similarity, and Flesch Reading Ease score were evaluated. |
| Data sources/ measurement | 8* | MCQ Preparation and Chatbot Assessment. MCQs were obtained from the Bootcamp repository and validated by three prosthodontic experts. Chatbot responses were compared with expert-validated ground-truth answers. |
| Bias | 9 | Standardized Evaluation Procedure. All chatbots received identical prompts and questions under standardized testing conditions. Independent chat sessions were used to minimize contextual memory effects and response variability. |
| Study size | 10 | MCQ Dataset Selection. A total of 150 MCQs were selected to provide broad coverage of major prosthodontic domains and enable comprehensive chatbot comparison. |
| Quantitative variables | 11 | Statistical Analysis. Quantitative metrics including accuracy, BLEU score, WER, cosine similarity, and readability scores were calculated and compared across chatbots. |
| Statistical methods | 12 | (*a*) Statistical Analysis. Descriptive statistics, 95% confidence intervals, ANOVA, and Mann-Whitney test were used to compare chatbot performance metrics. |
|  |  | (*b*) N/A – No subgroup or interaction analyses were performed. |
|  |  | (*c*) No missing data were observed; therefore, no imputation procedures were required. |
|  |  | (*d*) Sampling strategy adjustment was not applicable. |
|  |  | (*e*) Sensitivity analyses were not performed. |
| Results | | |
| Participants | 13* | (a) N/A – No human participants. A curated dataset of 150 prosthodontic clinical scenario-based MCQs was selected and analyzed. |
|  |  | (b) N/A – No participant recruitment or non-participation. |
| Descriptive data | 14* | (a) Characteristics of the MCQ dataset and chatbot performance metrics. |
|  |  | (b) No missing data were observed for any evaluated variable. |
| Outcome data | 15* | Accuracy, BLEU score, Word Error Rate (WER), cosine similarity, and Flesch Reading Ease scores reported for all chatbots. |
| Main results | 16 | (a) Performance estimates reported with 95% confidence intervals and comparative analyses. |
|  |  | (b)N/A – Continuous variables were not categorized. |
|  |  | (*c*) N/A – Relative risk measures were not applicable. |
| Other analyses | 17 | One-way ANOVA performed to compare BLEU score, WER, cosine similarity, and readability metrics among chatbots. |
| Discussion | | |
| Key results | 18 | Summary of the comparative performance of ChatGPT, Claude, Copilot, and DeepSeek in relation to the study objective. |
| Limitations | 19 | Limitations section discussing MCQ-based evaluation, model updates, and potential response variability. |
| Interpretation | 20 | Interpretation of findings with reference to previous AI and dental education literature. |
| Generalisability | 21 | Potential educational applicability of AI chatbots in prosthodontics with caution regarding clinical generalization. |
| Other information | | |
| Funding | 22 | Funding source and the role of the funder are provided in the Funding section. |
